# Communication from Charles Weil, M. D.

**Published:** 1885-05

**Authors:** Charles Weil

**Affiliations:** Buffalo


### Correspondence.

*Editors Buffalo Medical and Surgical Journal:*

In his comments on Dr. Charles G. Stockton's recent paper on "Some Untoward Effects of Opium," read before the Buffalo Medical and Surgical Association,* my friend, Dr. H. R. Hopkins,† referred to two cases of his own, in which "a peculiar condition followed immediately upon a hypodermic injection of morphia, a red glow coming upon every portion of the exposed skin, and in which the pulse became very greatly accelerated. The doctor believed the action was upon the nervous system, because these symptoms appeared before it was possible for the solution to have become absorbed, and almost before the needle had been withdrawn."

Your correspondent regrets that he was not able to have been present at the reading and discussion of this paper, because he also has had some interestingly peculiar effects produced in several patients by the hypodermatic administration of this priceless remedy, and for this reason he takes this method of recording, in as brief a manner as possible, what he would have said had he been able to attend this meeting.

Among these effects were precisely such as friend Hopkins described. The body, as far as seen, becomes red from the determination of blood to the surface, and some of the patients straightway began to scratch themselves at various parts, com-

* Thursday evening, January 6, 1885.

† *Vide* report of the proceedings as published in this Journal, current issue, p. 365.

plaining of itching, particularly marked on the face and palms of both hands. In some of these cases there was a pronounced itching of the lining membrane of the nose also, as evinced by an effort of the patients to get at it to scratch, and by the sneezing it produced even before they got to scratch themselves. The pulse became almost uncountable, and in some instances quite so; the respiration also, for a few seconds, seemed to be much embarrassed, but within a few minutes all these unpleasant and more or less alarming symptoms passed off, including likewise the buzzing in the ears and the throbbing in the head, which some complained of, in addition in three or four cases.

These effects, be it remembered, were produced, as in Dr. Hopkins' cases, before the withdrawal of the needle, indeed, twice before the syringe had been emptied, the injection having been slowly, but steadily, made, *i. e.*, without interruption, by continuous action.

Now, and this is the point it is desired to bring out, these symptoms occurred only when a vein was struck. When they were first observed the writer could not account for their appearance; but when one day a vein was entered for the purpose of producing as quick an action of the drug as possible, because the patient was suffering most excruciatingly and outrageously, and when these effects thereupon made their appearance, to make sure that the explanation, which then suggested itself, was correct, the needle was designedly inserted in veins in some of the cases in which it had been necessary to resort to morphia hypodermically, always with the phenomena described produced almost instantaneously. The writer now regrets also that he did not make memoranda of the names, dates, etc., as further evidence of the facts here stated. But these demonstrations have been done in a sufficient number of instances to warrant these simple statements, and the immediate cause of the phenomena observed. In no case, in which a blood vessel was struck or entered, were any of those symptoms produced, where, as whenever a blood vessel had been entered and the solution injected into it, they never failed to appear, either wholly or in part, and mo re or less pronounced, according to the strength of the solution or the idiosyncracy of the patient, as a matter of course. And as an evidence, in not a few cases at any rate, that a vein had been entered, several drops of venous blood would follow the withdrawal of the needle, or if the piston was drawn up while the needle was still *in situ*, blood was drawn up into the syringe. I am well aware of the importance of avoiding a vein, *because of the sudden, overwhelming effect*, and would not recommend its adoption. In the cases in which veins were not avoided, the greatest care was exercised to inject slowly, though steadily.

The explanation of Dr. Hopkins that it is through the nervous system that these symptoms are produced, is concurred in in so far as we can call them the remote effects of the drug. We all know that nitro-glycerine and amyl-nitrate produce similar symptoms through their action on the vaso-motors, and it is in this way, doubtless, that morphia acts.

But in so far as the doctor meant that the morphia acted on the nervous system instanter, "before it was possible for the solution to have become absorbed;" in so far as he means by this that these symptoms were the result of the immediate action of the morphia on the nervous system without having been carried to the nervous system by absorption into and then by the blood, or by the blood right off, because thrown into it—from that opinion I would most respectfully dissent. No remedy can produce a systemic or constitutional effect without having entered, through the process of absorption, the blood, and without its presence in the blood, a few drugs excepted, of which, however, morphia is not one. (*Vide* for example, Headland, "Art of Med.," Props. I. and V. especially.

Therefore, these peculiar symptoms were produced by the action of the morphia on the vaso-motors, by or through its instantaneous carriage to this system of nerves by the blood. Absorption was, as a matter of course, not necessary, there having been done artificially what absorption does naturally—carry the drug to the blood for circulation in it. And reference has already been made to the fact that when thrown directly in the blood, the effect of the morphine is sudden and overwhelming, for which very reason, as already stated, this should not be done. The cases cited here, and this fact explains, I believe, the rationale of these symptoms. If thrown into the subcutaneous cellular tissue, it is slowly absorbed and slowly carried to the blood; and hence there is no overwhelming effect as is produced by its direct injection into the blood, which manifests itself in the symptoms described by the doctor and myself.

It is proper to add, in conclusion, that what is here expressed is simply and solely my own deductions, and my experimentations, if so we wish to call my desire to find out whether my first impression was right, ought to be worth something, inasmuch as I have demonstrated this question in no less than at least ten or eleven instances. But what is here written was not prompted by any wish to be critical, but simply by a desire to throw light, if possible, upon this point, since no one seems to have taken any exceptions to the explanation of Dr. Hopkins, while the matter is certainly of sufficient importance to merit considerable attention.

CHARLES WEIL, M. D.

BUFFALO, March, 1885.

